# Long distance signalling and epigenetic changes in crop grafting

**DOI:** 10.3389/fpls.2023.1121704

**Published:** 2023-03-20

**Authors:** Katie Jeynes-Cupper, Marco Catoni

**Affiliations:** ^1^ School of Biosciences, University of Birmingham, Birmingham, United Kingdom; ^2^ Institute for Sustainable Plant Protection, National Research Council of Italy, Torino, Italy

**Keywords:** solanaceous, cucurbitaceous, epigenetic, DNA methylation, grafting, mobile siRNA, signaling molecules, graft junction

## Abstract

Humans have used grafting for more than 4000 years to improve plant production, through physically joining two different plants, which can continue to grow as a single organism. Today, grafting is becoming increasingly more popular as a technique to increase the production of herbaceous horticultural crops, where rootstocks can introduce traits such as resistance to several pathogens and/or improving the plant vigour. Research in model plants have documented how long-distance signalling mechanisms across the graft junction, together with epigenetic regulation, can produce molecular and phenotypic changes in grafted plants. Yet, most of the studied examples rely on proof-of-concept experiments or on limited specific cases. This review explores the link between research findings in model plants and crop species. We analyse studies investigating the movement of signalling molecules across the graft junction and their implications on epigenetic regulation. The improvement of genomics analyses and the increased availability of genetic resources has allowed to collect more information on potential benefits of grafting in horticultural crop models. Ultimately, further research into this topic will enhance our ability to use the grafting technique to exploit genetic and epigenetic variation in crops, as an alternative to traditional breeding.

## 1 Introduction

The global population is anticipated to grow to around 9.9 million by 2050, and with that, food production will need to at least double to meet the increasing demands (World population data sheet 2020, https://interactives.prb.org/2020-wpds/). This is of particular concern considering that arable land dropped by more than half from 1961 to 2016 as a consequence of housing and industrial needs, as well as excessive agrochemical usage. One of the most obvious solutions to this problem is to increase crop productivity, which can be obtained by breeding more favourable traits or using more productive agronomical techniques. Plant grafting is an ancient horticultural technique traditionally used on cultivated trees which has more recently (since the late 1920s) been exploited as a system to introduce resistance and improve productivity in horticultural herbaceous crops (Tateishi, 1927; Goldschmidt, 2014; Gaion et al., 2018). This technique involves joining two plants together, where one contributes to the upper part (scion) and the other contributes to the lower parts, including the roots (rootstock). In compatible combinations, the scion and the rootstock will grow together as a single plant (Mudge et al., 2009). Due to its simple application, this method is agronomically sustainable and has had many positive effects on food security. The most memorable use of grafting in agriculture involved the introduction of resistance to the soil-borne insect phylloxera (*Phylloxera vastatrix*) in the European wine industry in the 19^th^ century (Pouget, 1990).

Until the 20^th^ century, grafting was limited to woody species but has since been extended to herbaceous high-value crops (Lee and Oda, 2002; King et al., 2010). Research on herbaceous plant grafting was first described in a study in 1927, which found that watermelon (*Citrullus lanatus*) plants grafted onto pumpkin (*Cucurbita* spp.) were more resistant to pathogens and had a higher fruit yield (Tateishi, 1927). By the mid 1990s, grafting of *Arabidopsis thaliana* had been extensively exploited to understand various phenomena, including the movement of nutrients between the roots and shoots and the study of specific gene functions (Tsutsui and Notaguchi, 2017). The most grafted herbaceous crops with commercial relevance belong to the *Solanaceous* or *Cucurbitaceous* family which are grafted to increase their production and to transfer favourable traits from unproductive species or genotypes.

From 2000 to 2020, three regularly grafted species, tomatoes (*Solanum lycopersicum*), eggplant (*Solanum melongena*) and cucumber (*Cucumis sativus*), accounted for 42-45% of the total increase of the global vegetable production (FAO, 2022). This shows that the global cultivation of these crops is of high economic importance, and the use of grafting could contribute to improving their production. In this context, many seed companies have developed programs to breed rootstocks to improve the yield of these crops (Goldschmidt, 2014). These rootstocks are selected for specific traits, such as the ability to provide drought tolerance, soil-borne pathogen resistance, or scion vigour. Some effects of grafting can be explained simply by the intrinsic characteristics of the genotypes involved. For example, a rootstock can provide resistance to soil-borne pathogens due to their natural immunity, preventing the pathogen from accessing the aerial plant portion. However, it is unclear why a rootstock (or scion) can introduce a trait to a particular grafting partner, depending on the combination of genotypes used. This effect mainly involves scion vigour and changes in plant architecture, making it challenging to predict the outcome of grafting between the combination of two genotypes. It is commonly accepted that substances such as hormones, proteins and signalling molecules can be translocated between the scion and rootstock, and can impose a large biological effect on the recipient tissue (Haroldsen et al., 2012). Additional studies have proposed that such signalling molecules can introduce epigenetic changes that drive grafting-induced phenotypes (Molnar et al., 2010; Lewsey et al., 2016). Molecular alterations in the scion can ultimately change the transcriptome and plant phenotypes, thereby controlling critical aspects of plant development (as reviewed by Feng et al., 2010). Although these effects have been observed in proof-of-concept studies mostly in *Arabidopsis thaliana*, for relevant agronomical traits we often lack direct evidence of the translation of such observations into crops. In this review, we summarise our understanding of the movement of signalling molecules that can trigger epigenetic effects in plant grafting and discuss the role of epigenetics in producing favourable horticultural traits in grafted crops and their progeny.

## 2 Phenotypic traits and transcriptomic alteration in crop grafting

Over the past decade, researchers have discovered a link between the introduction of grafting-induced phenotypic traits and changes at the transcriptional level. For example, cold-tolerance in tomatoes can be transferred to a susceptible scion by grafting it onto a cold-tolerant tomato rootstock, and this tolerance is associated with changes in the expression of defence-related genes (Ntatsi et al., 2017). Similarly, in watermelon, scions grafted onto bottle gourd (*Lagenaria siceraria*) rootstocks have been shown to trigger an increase in scion fruit size and rind thickness (Garcia-Lozano et al., 2020). Additionally, the improved scion phenotype has been linked to the differential expression of genes related to ripening, softening, cell wall strengthening, stress response and disease resistance (Garcia-Lozano et al., 2020). Interestingly, in the reverse grafting combination (using melon as a rootstock and bottle gourd as the scion), the produced fruits had a higher soluble solid content and thinner rinds (Garcia-Lozano et al., 2020). A previous study on watermelon grafted plants showed that productivity is strongly linked to the efficiency of water uptake (Rouphael et al., 2008). However, it is unclear whether this improved use of water is a consequence of the intrinsic propriety of the rootstock (e.g. extended root system) or the result of induced transcriptional changes.

In an experiment where tomatoes were grafted onto potatoes (*Solanum tuberosum*) to introduce late blight resistance, differences in the transcriptome were observed in both the scion and rootstock of hetero-grafted plants compared to self-grafted controls; however, it was unclear whether such alterations were simply due to changes in photosynthates production (Li and Zhao, 2021). Transcriptional alterations observed in grafted plants have not always been associated with phenotypical changes. In a study where watermelon was grafted onto bottle gourds or squash (*Cucurbita* spp.), the authors identified changes in the expression of many genes in the scion, but they did not observe phenotypic alterations between the heterografted and self-grafted plants (Liu et al., 2016). Similar results were obtained in a study on tomatoes, where heterografted scions displayed altered transcriptional profiles for genes related to oxidative stress, but in absence of phenotypic differences (Wang et al., 2019).

The study of the link between the phenotype and transcriptome in grafted plants is complicated by the fact that the grafting method itself can cause transcriptional changes in scions, as observed in experiments performed with Arabidopsis and tomato self-grafted plants (Kumari et al., 2015; Spanò et al., 2015). Studies performed in combination with pathogen viral infections have also suggested that resistant or tolerant phenotypes could be due to a combination of the grafting method and the interactions between rootstock and scion. For example, a study examining the recovery from infection by *Potato virus Y* (PVY) in tomatoes demonstrated that the grafting technique itself improved recovery from the infection regardless of the use of either a susceptible or resistant rootstock (Spanò et al., 2020). Similarly, a report on the transmission of *Cucumber mosaic virus* (CMV) resistance by grafting in tomatoes showed that grafting a susceptible variety onto a resistant variety would enable the same disease recovery observed in the resistant lines, but recovery was also observed in the self-grafts of the susceptible variety (Spanò et al., 2017). Viruses move through the plant vascular tissue and must eventually pass through a graft junction to establish a systemic infection; thus, the alteration of vascular connections induced by grafting can directly affect the spread of the virus. Nonetheless, a transgenic tomato rootstock producing viral sRNAs and resistant to the *Tomato spotted wilt virus* (TSWV) was found to delay symptoms and strongly reduce virus accumulation in grafted scions, compared to scions grafted on susceptible tomato lines (Catoni et al., 2013). This indicates that a certain degree of tolerance to viral infection mediated by viral-derived sRNAs can, at least in certain instances, be transferred through grafting. Collectively, we have good evidence that grafting of different genotypes induces changes in gene expression, although this is not always associated with a clear transfer of a phenotype.

## 3 Movement of RNAs across the graft junction

Plants have evolved complex communication networks and transport systems, where roots and shoots constantly communicate to coordinate growth and development (Liu and Chen, 2018). Such communication is facilitated by the vascular system, which comprises of the xylem and phloem. The xylem is responsible for the transportation of inorganic salts and water, while the phloem is responsible for the transportation of plant hormones, photosynthates, amino acids and notably mobile RNA molecules such as messenger RNA (mRNA), small interfering RNA (siRNA) and micro RNA (miRNA) (Hu et al., 2016). When plants are grafted, the vascular systems of the scion and rootstock fuse in a compatible match. The phloem is the first to connect, followed by the xylem, along with the formation of plasmodesmata between adhered cells, which establishes cell-to-cell communication and facilitates long-distance signalling (Jeffree and Yeoman, 1983; Kollmann and Glockmann, 1985; Ferreres et al., 2011; Melnyk et al., 2015).

Mobile RNAs are well known to be transported inside plants and act as signalling macromolecules to coordinate an array of physiological processes (Lucas et al., 2001; Lough and Lucas, 2006; Luo et al., 2018). Grafting in *Arabidopsis thaliana* has been used extensively to prove that RNAs can travel bidirectionally between rootstock and scion tissues *via* the grafting junction (Harada, 2010; Spiegelman et al., 2013; TurnbullLopez-Cobollo, 2013). Some mobile RNAs can introduce epigenetic changes to their destination tissues and trigger alterations in plant physiology and architecture (Kehr and Kragler, 2018; Liu and Chen, 2018). Numerous studies have demonstrated that also non-coding RNAs can regulate the expression of specific genes *via* transcriptional repression playing an important role in the emergence of specific phenotypes or traits in response to development or stress. Such transcriptional repression is achieved *via* changes in the epigenome, specifically DNA methylation (Song et al., 2003; Zhang et al., 2018a; Zhang et al., 2018b).

### 3.1 Movement of small non-coding RNA molecules

**Figure 1:**
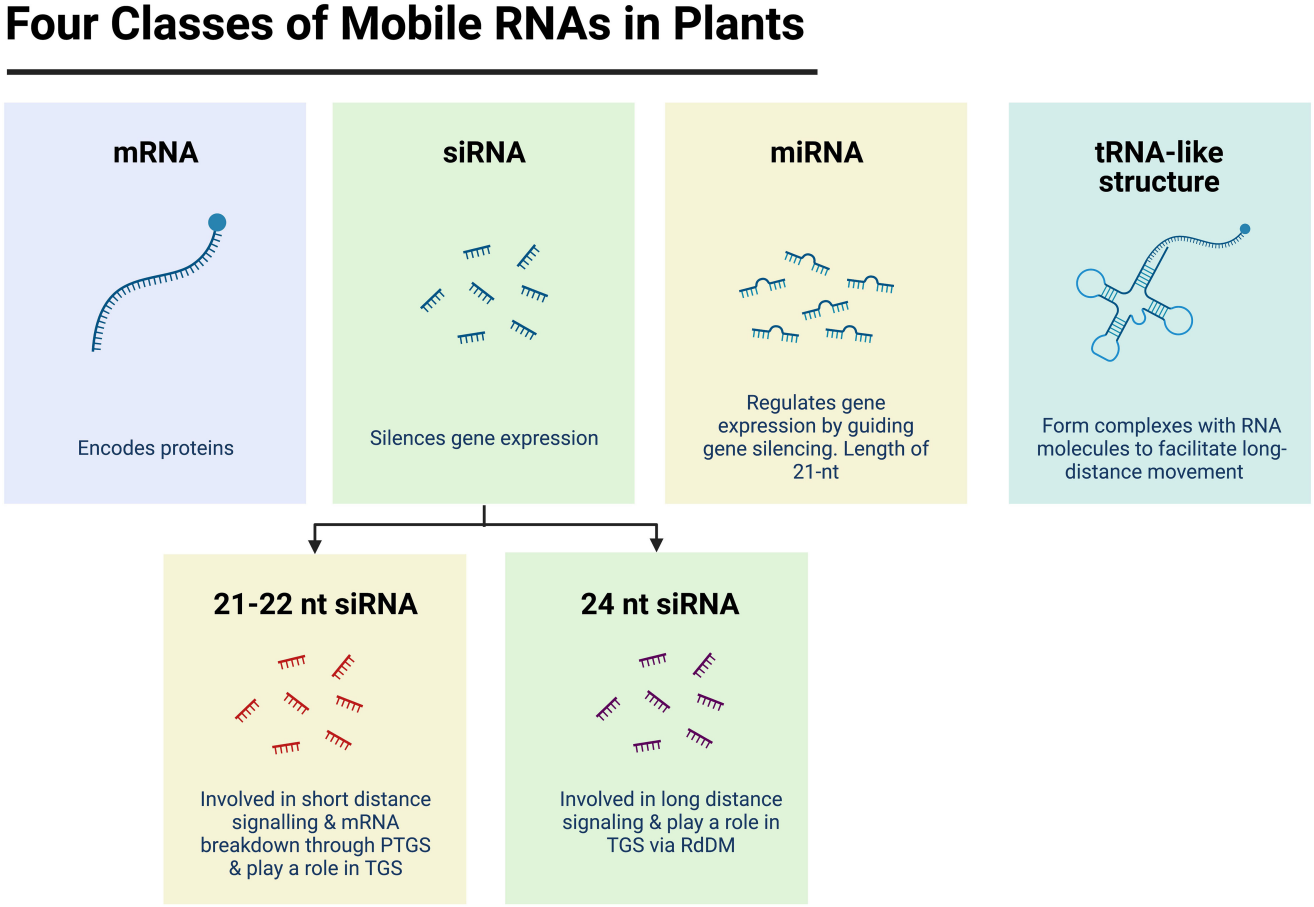
Four classes of mobile RNA molecules in plants. Potential mobile RNA molecules include messenger RNA (mRNA) molecules which encode proteins; transfer RNA (tRNA) -like structures which are associated to movement of RNA molecules; micro RNA (miRNA) which regulate gene expression by guiding gene silencing mechanisms such as Post Transcriptional Gene Silencing (PTGS) and small interfering RNAs (siRNA) which mediate the silencing of gene expression through PTGS and/or through Transcriptional Gene Silencing (TGS) *via* the RNA-directed DNA methylation pathway (RdDM). Figure created with BioRender.com.

Small non-coding RNAs represent a cohort of different molecules in plants, with a size of 20 – 24 nucleotides. They can be broadly classified into two groups: i) Micro RNAs (miRNA), or ii) Small interfering RNAs (siRNA or sRNA). These two classes differ by the way siRNAs are synthesised and how they operate (Borges and Martienssen, 2015; Kamthan et al., 2015; Singh et al., 2018). In plants, miRNAs are encoded by endogenous genes that are transcribed into long primary miRNAs (pri-miRNAs) that are characteristically folded into a hairpin-like structures. These are then processed by RNase III-like Dicer 1 (DCL1), first into stem-loop structures, known as precursor miRNAs (pre-miRNAs), then into mature miRNA duplexes. The resulting miRNAs can prevent mRNA translation through Post Transcriptional Gene Silencing (PTGS), which ultimately causes gene repression (Borges and Martienssen, 2015) (**Figure 1**). In comparison, siRNAs are derived from long double-stranded RNA precursors and are grouped based on their function. The first group contains siRNAs with a nucleotide length of 21 - 22 and are derived from the activity of Dicer-like 2 and Dicer-like 4 (DCL2/DCL4) from trans-acting small interfering RNA (TAS) genes, transcripts or other aberrant RNA molecules (Berger et al., 2018). The 21/22-nt siRNAs are known to be involved in short-distance signalling and in the degradation of mRNAs (Tan et al., 2020), as well as in targeted Transcriptional Gene Silencing (TGS) (**Figure 1**) (Borges and Martienssen, 2015). The second group is known as 24-nt siRNAs which originate from heterochromatic regions by the continuous action of three enzymes: RNA Polymerase IV, RNA Dependent RNA polymerase 2 (RDR2) and Dicer-like 3 (DCL3). The 24-nt siRNAs are renowned for their involvement in the RNA-directed DNA methylation (RdDM) pathway by guiding the Domain rearranged DNA methyltransferases (DRMs) in TGS, but they are also known for their involvement in long-distance signalling (Berger et al., 2018) (**Figure 1**).

**Figure 2:**
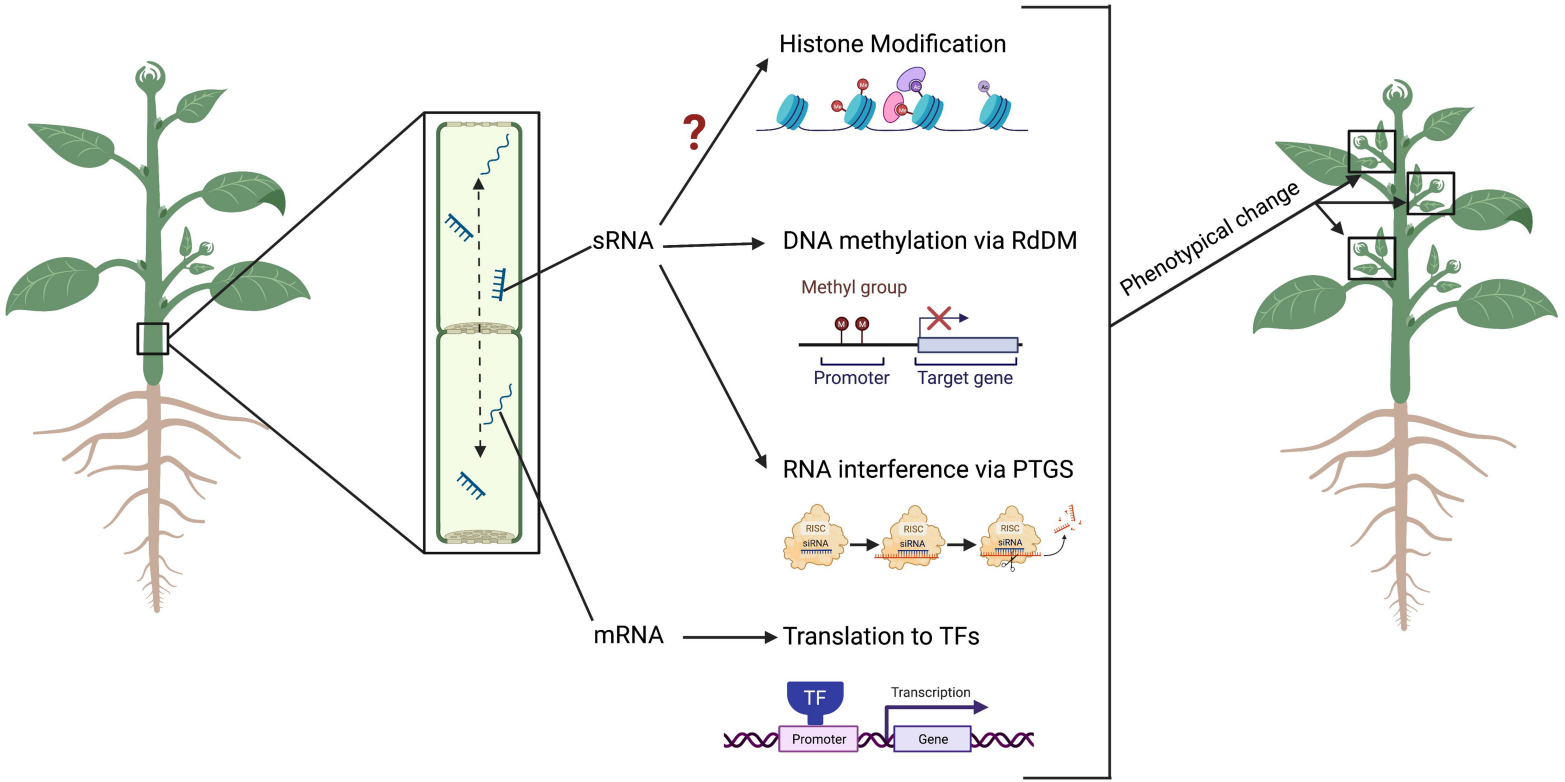
The movement of small RNA (siRNA) between root and shoot drive epigenetic modifications and lead to phenotypical changes in the plant. The siRNAs can move both from root-to-shoot and shoot-to-roots directions, *via* the phloem through the sieve tubes. Once at the destination tissue, the siRNAs can alter DNA methylation at specific loci *via* the RNA-directed DNA Methylation (RdDM) pathway, or prevent the translation of a gene product *via* the Post-Transcriptional Gene Silencing (PTGS) pathway. Messenger RNAs (mRNA) can move across a systemically (and across a graft junction) into cells where they are translated into transcription factors (TFs) to control gene expression. Although not yet demonstrated in grafting experiment, siRNAs can introduce histone modifications. Alterations to these epigenetic marks can result in changes to the plant phenotype including resistance to abiotic and biotic stress, as well as alteration in the plant architecture and vigour. Figure created with BioRender.com.

In the model species *Arabidopsis thaliana*, it has been demonstrated that the systemic movement of siRNAs can direct *de novo* DNA methylation in tissues distant from their origin (Borges and Martienssen, 2015; Xie and Yu, 2015) (**Figure 2**). This involves the recruitment of DNA methyltransferases in recipient cells or tissues, and the loading of the siRNA into ARGONAUTEs (AGOs) proteins, which are then recruited into a complex with transcribed scaffold RNA by the action of RNA Polymerase V (Huang et al., 2021). More specifically, AGO1 protein in Arabidopsis has been proposed to remove sRNAs from traversed tissue, allowing their accumulation in recipient cells, a phenomenon defined as “consumption” of the mobile sRNAs (Denvers et al., 2020). Further studies identified in Squash pumpkin (*Cucurbita maxima*) phloem exudates a set of specific proteins able to bind sRNAs and mediate non-cell autonomous movement of both 21/22- and 24- nt classes (reviewed in Yan and Ham, 2022). Among these, the PHLOEM SMALL RNA-BINDING PROTEIN1 (PSRP1) has being found able to form a ribonucleoprotein complex in the phloem sieve tube system (Ham et al., 2014), while the SMALL RNA-BINDING PROTEIN 1 (SRBP1) homologues in Arabidopsis has been found capable of cell-to-cell movement and has a role in sRNA trafficking into the apoplast (Yang et al., 2019; Karimi et al., 2022). These results suggest the existence of a specific mechanism for the systemic mobilization of sRNA molecules in plants, which is currently subject of research and discussion (Voinnet, 2022; Yan and Ham, 2022).

The translocation of siRNAs across the grafting junction has also been explored in several crops, including eggplant, pumpkin (*Cucurbita pepo)*, cucumber, potato, *Nicotiana benthamiana*, grapevine (*Vitis vinifera)* and several bean (*Fabaceae*) species (Tournier et al., 2006; Bai et al., 2011; Zhang et al., 2014; Cerruti et al., 2021; Li et al., 2021; Davoudi et al., 2022; Rubio et al., 2022; Zhang et al., 2022). In addition, several miRNAs in potato have been demonstrated to be graft-transmissible, including *miR156*, *miR339*, *miR395* and *miR172* (Pant et al., 2008; Buhtz et al., 2010; Kasai et al., 2010). *MiR156* has been found to influence plant architecture and tuberization by targeting the regulation of specific genes (Bhogale et al., 2013). The movement of siRNAs was also studied by Kasai et al., who demonstrated that artificial siRNAs in *N. benthamiana* scions could travel into the potato rootstock to induce TGS *via* RdDM on both a transgene and an endogenous gene (Kasai et al., 2016). In a recent report, grafting was used to induce editing in scions using clustered regularly interspaced short palindromic repeats (CRISPR)-associated protein 9 (*Cas9)* and guide RNA transcripts moved from a transgenic rootstock (Yang et al., 2023). In addition to demonstrating the potential use of grafting in combination with genome editing, this study also showed that mobilised transcripts in the scion can accumulate at a sufficient abundance to introduce a gene editing effect.

In plants, the PTGS pathway falls under RNA interference (RNAi) (Ashfaq et al., 2020). This mechanism involves the suppression of gene expression through degradation of mRNA transcripts *via* the production of siRNAs (Ashfaq et al., 2020) (**Figure 2**) (Hamilton and Baulcombe, 1999; Jones et al., 1999; Mette et al., 1999; Dalmay et al., 2000; Marjori et al., 2001). Therefore, it is accepted that both PTGS and DNA methylation function cooperatively or in a dependent manner as initially suggested by Morel et al., 2000 (Morel et al., 2000), and subsequentially proved in multiple experimental conditions (Palauqui et al., 1997; Sonoda and Nishiguchi, 2000; Voinnet et al., 2000; Crété et al., 2001; Mallory et al., 2001; Mallory et al., 2003; García-Pérez et al., 2004; Hewezi et al., 2005; Brosnan et al., 2007; Brumin et al., 2009; Kasai et al., 2011; Taochy et al., 2017; Chen et al., 2018). However, current literature exploring this aspect in grafted plants is limited, and phenotypic changes induced by grafting have only been indirectly linked to DNA methylation alterations (Cerruti et al., 2021).

### 3.2 Movement of mRNA transcripts

Unlike siRNAs, mobile mRNAs can directly contribute to the synthesis of proteins into recipient tissues. They have been found to be particularly relevant as transcription factors (TFs), which play a key role in controlling the expression of several genes in a specific pathway. The long-distance mobilisation of mRNAs has been documented in several models using grafting as a tool. For example, in tomato it has been observed that the mRNA transcript *PFP-LeT6*, composed by a *P*YROPHOSPHATE-DEPENDENT PHOSPHOFRUCTOKINASE (PFP) and tomato KNOTTED-1–like homeobox (KNOX) domain (LeT6), is able to travel from roots to shoots and induce a change in the leaf morphology (Kim et al., 2001). Similarly, both RT-PCR and *in situ* hybridization revealed that mRNA transcripts of two partner transcription factors, the *potato homeobox1* (*POTH1*) gene and BEL1-type *StBEL5*, can move across the graft junction and regulate hormone accumulation, alter leaf architecture, and boost tuber formation (Banerjee et al., 2006; Mahajan et al., 2012; Sharma et al., 2014). Additional studies on pumpkin grafts have also identified other mobile mRNA transcripts that are likely to drive molecular and phenotypic changes, including those encoding transcriptional regulators of auxin signalling (Omid et al., 2007). This was followed by studies on *Arabidopsis*, which characterised specific auxin mRNA transcripts produced in leaf tissue and translocated to the root system, where they negatively regulate lateral root formation (Omid et al., 2007; Notaguchi et al., 2012). Within the same timeline, researchers have also identified similar hormone-related mRNA transcripts in tomato, cucumber, and pumpkin (Kim et al., 2001; Haywood et al., 2005; Banerjee et al., 2006; Notaguchi et al., 2012; Davoudi et al., 2022). In *Arabidopsis*, the dwarf phenotype observed in gibberellin (GA)-deficient mutants can be restored if plants are grafted onto WT (Magome et al., 2004), whereas tomato GA-deficient rootstocks can introduce a dwarf-like phenotype in the scion, which is mediated by the movement of transgenic mRNA transcripts up the graft junction (Wang et al., 2012). In tomato has been also found that the mRNA encoding for the GIBBERELLIC ACID INSENSITIVE (GAI) factor produced in a rootstock can transcriptionally regulate gibberellic acid response genes within the scion tissue (Haywood et al., 2005). In addition, there has been evidence that a GAI homologous can be transported in pumpkins by an RNA-protein complex (Ham et al., 2009). In a study investigating drought tolerance in cucumber and pumpkin (*Cucumis moschata*) grafts, authors identified that the movement of mobile mRNAs correlated with significant accumulation of abscisic acid (ABA) from the pumpkin rootstock in the cucumber scion tissue (Davoudi et al., 2022).

Collectively, it has been well-documented, both in model and crop species, that at least some mRNAs can move across the graft junction and induce phenotypic and developmental changes. However, it remains unclear whether these transcripts are primarily responsible for the observed traits or instead trigger more general epigenetic or post-transcriptional regulation mechanisms responsible for the scion/rootstock physiological response. Hence, further work could be directed to mechanistically associate mobile signalling molecules to an observed epigenetic and/or phenotypic change in grafted plants. In this context, the use of experimental grafting associated with genome-wide sequencing approaches appears to be a powerful tool for correlating phenotypes to mobile mRNA molecules. In experiments where scions and rootstock were from different sequenced genomes, it became possible to compare mRNA sequencing experiments with DNA sequence variants in the two genotypes and identify mobile mRNAs with greater confidence. The methods used to analyse transcriptomic data are well established in plants, but only few analyses or pipelines have been specifically designed for the detection of mobile transcripts (Tomkins et al., 2022). Further development of such tools should be considered to support a better understanding of the molecular mechanisms underlying grafting-induced traits.

### 3.3 Transport mechanisms

The transport mechanism of mobile RNAs is still not well understood and has been predominantly investigated in the model species *Arabidopsis thaliana*. One of the first studies to identify an association between mRNA abundance and mobility in grafted plants proposed a hypothesis for RNA transport based on passive movement (Thieme et al., 2015). This was supported by a more recent study that found that long-distance movement of mRNA is linked to their local abundance (Calderwood et al., 2016). However, this work could only explain the movement of some mRNAs, and further hypotheses suggest the simultaneous passive and active movement of mobile RNA species (Kim et al., 2014; Notaguchi et al., 2015). The active transportation of RNA molecules has been then proposed in multiple subsequent studies (Zhang et al., 2009; Thieme et al., 2015; Zhang et al., 2016; Garcia-Lozano et al., 2020). Such active movement appears to be associated with the presence of transfer RNA-like structure (TLS) motifs, which can mediate the mobility of some mRNA transcripts to distant locations, as observed in both *Arabidopsis* and pumpkin (Zhang et al., 2009; Zhang et al., 2016). Experimental work has shown that the addition of a TLS motif, or a specific sequence derived from a TLS motif, is sufficient to introduce transcript mobility, while their removal from an RNA molecule prevents long-distance transportation (Garcia-Lozano et al., 2020; Yang et al., 2023). Collectively, these results suggest that TLSs or related structures may control the translocation of mobile transcripts in graft systems.

In addition to sequences associated with mobility in RNA molecules, also cytosine methylation can play a role in RNA mobility (Yang et al., 2019). Both plant phloem transfer RNAs (tRNAs) and graft-mobile mRNAs contain TLS motifs that have a high probability of being targeted by RNA methyltransferases (Yang et al., 2019). Hence, mobile RNA transcripts are enriched in cytosine methylation, and if such methylation is removed, mobility can be negatively affected (Yang et al., 2019). Much of what is known about the transport of RNA species in plants is credited to research conducted on viroids, which are circular non-coding RNA species often considered plant pathogens, mostly because of their recognition as an ideal model system for molecular transport studies (Capistrán-Barradas et al., 2006; Ding and Itaya, 2007; Wang et al., 2021). For example, research performed on viroids has found that their long-distance mobility is facilitated by selective trafficking based on the presence of RNA motifs or structures (Qi et al., 2004; Zhong et al., 2007; Zhong et al., 2008; Takeda et al., 2018) and a similar concept has been supported by several studies related to plant-derived mobile RNAs (Zhang et al., 2009; Thieme et al., 2015; Zhang et al., 2016; Garcia-Lozano et al., 2020). In addition, experiments performed with grafting in cucumber demonstrated that viroid movement is mediated by specific host proteins (Gómez and Pallás, 2004), suggesting that a similar mechanism exists for endogenous RNA molecules. Interestingly, it was found that a specific plant protein assists the mobility of at least one endogenous mRNA in pear (*Pyrus betulaefolia*) (Duan et al., 2016), and recent studies have suggested that organelle-assisted movement is also used for the translocation of endogenous sRNAs in herbaceous plants (Luo et al., 2022). This hypothesis suggests that mRNAs move by interacting with organelles that are transported out of the phloem into the destination tissue (Luo et al., 2022). An example of this mechanism was observed with the mRNA of the mobile factor FLOWERING LOCUS T, which has been found to move through the plasmodesmata *via* microtubules in the endosomes (Luo et al., 2022). A similar finding was observed in maize, where mobilisation of the mRNA of the KNOTTED1 (KN1) TF, a highly conserved protein involved in stem cell production, moves in shoot meristems. This is of particular interest because, although the movement of TFs is widely known, for KN1 functionality it requires trafficking of both its protein and mRNA (Kitagawa et al., 2022).

Collectively, the data indicates the existence of active mechanism for the systemic transport of at least one group of mobile RNA molecules. Considering that distant mobile molecules can trigger effects through a graft junction, these studies are important for clarifying the mechanisms underlying how a rootstock can induce physiological changes in scions or vice versa.

## 4 Changes to the epigenome associate to grafting

Epigenetic marks are constituted by molecular alterations of chromatin which does not alter the sequence of the DNA itself, and can play critical roles in controlling the expression of genes, with consequences on the morphology, physiology, and ecology of many organisms (Rapp and Wendel, 2005; Fossey, 2009). In plants, there are two main categories of epigenetic marks: DNA methylation and histone modifications (Rapp and Wendel, 2005; Fossey, 2009). Cytosine is the only DNA nucleotide that is methylated, and there are a multitude of different histone modifications, including acetylation, methylation, phosphorylation, ribosylation, and ubiquitination (Pfluger and Wagner, 2007; Liu et al., 2010). These epigenetic changes play a role in the regulation of chromatin structure and control the accessibility of DNA (Robertson, 2005; Lang et al., 2017). Epigenetic variation can generate epigenetic alleles (or epialleles) with different degrees of stability, which are transmitted during DNA replication and often mitotically passed to progeny in a Mendelian fashion (Weigel and Colot, 2012; Catoni and Cortijo, 2018). In the previous chapter, we discussed how mobile RNA molecules travelling across the graft junction can trigger epigenetic changes at specific target loci in recipient tissues. Therefore, in this section, we discuss the evidence of epigenetic alterations associated with plant grafting.

### 4.1 Alterations of DNA methylation in grafted plants

Changes in DNA methylation associated with grafting have been observed in many plant families. The first studies were conducted on *Brassicaceae*, *Cucurbitaceae*, and *Solanaceae* species (Molnar et al., 2010; Avramidou et al., 2015; Lewsey et al., 2016). In *Arabidopsis*, a siRNA molecule originating from a transgene was found to move across the graft junction with prevalent translocation in the shoot-to-root direction. At specific loci, these molecules have been found to direct DNA methylation, resulting in a silencing signal (Molnar et al., 2010). The use of a transgenic construct has also been employed in tobacco to show that siRNA produced in the rootstock can travel across the graft junction and induce silencing of complementary DNA sequences in the scion by increasing DNA methylation (Bai et al., 2011). It was later revealed that the RdDM pathway has a critical role in triggering silencing by introducing DNA methylation at specific genomic loci (Lewsey et al., 2016). A general pattern of grafting-induced hypermethylation has been observed in *Cucurbitaceae* species when cucumber and melon were used a scion, grafted onto pumpkin rootstocks (Avramidou et al., 2015). In eggplant, however, hypomethylation in the scions was found to be associated with heterografting and linked to enhanced vigour (Cerruti et al., 2021). Similar studies across *Brassicaceae* and *Cucurbitaceae* identified methylation changes, mostly related to the non-CG context, associated with specific grafting combinations, but with different trends of hyper- and hypo-methylation (Avramidou et al., 2015; Lewsey et al., 2016; Cerruti et al., 2021). In these studies, the DNA methyltransferases and DNA demethylase genes in the scions were not found to be substantially differentially expressed, indicating that the epigenetic changes observed cannot be simply explained by altered expression levels of these epigenetic factors. Therefore, most of the DNA methylation changes associated with grafting could indeed be a consequence of a change in the siRNA targeting system or, alternatively, the result of other indirect effects of grafting (e.g. passive de-methylation or carry-over effects from the grafting procedure) (Wu et al., 2013; Cerruti et al., 2021).

Notably, hypomethylation and hypermethylation are associated with opposite effects on gene expression, and different combinations of scion and rootstock genotypes can lead to different phenotypic effects. Therefore, epigenetic changes could potentially explain part of the phenotypic alteration (e.g. vigour) observed in scions grafted onto different rootstock genotypes (Yang et al., 2018). In this context, it would be interesting to test the role of epigenetics in other grafting-induced phenotypes, such as disease resistance, which has been directly associated with epigenetic variation (Wada et al., 2004).

### 4.2 Grafting-induced histone modifications

While direct evidence of grafting-induced histone modifications has not yet been observed, there are studies suggesting that epigenetic modification induced by grafting is not limited to the alteration of the DNA methylation landscape (**Figure 2**). It is well established that there is a crosstalk between DNA methylation, sRNAs accumulation and histone modifications, so that epigenetic changes induced by distant signaling are expected to occur also at level of chromatin structure (Saze et al., 2012).

In a study performed on grapevine heterografts, the vigorous phenotype observed with specific grafting combinations was associated with the overexpression of the core histone genes as well as two nucleosome-associated proteins (Cookson and Ollat, 2013), while on apple tree varieties has been found that the expression of genes encoding histone modification factors was central to control flowering (Fan et al., 2018).

Therefore, histone modifications might play a role in the physiological effects induced by grafting and the extension of specific approaches to study chromatin dynamic (e.g. ATAC-seq or 3C) to grafting experiments could reveal more details on how histone marks can be associated with crop traits.

### 4.3 Transgenerational inheritance of grafting-induced epigenetic changes and implication to breeding

Epigenetic modifications were originally thought to only affect an individual during their lifespan and were not perceived to impact the offspring. In 1956, these beliefs were challenged and ideas that traits induced by the environment could be inherited began to be discussed (Waddington, 1956). Recently, epigenetic inheritance has shown that environmentally controlled epialleles can be transmitted to progeny, bridging the gap between inheritance by genetic determinants and environmental influences (Van Otterdijk and Michels, 2016; Pang et al., 2017). Hence, determining whether grafting-induced epigenetic alterations are transgenerationally inherited is pivotal to better understand plant developmental mechanisms and to exploit their potential use in breeding schemes to consistently increase crop production. In *Brassicaceae* models, DNA methylation alterations induced by grafting in the scion was shown to be indeed heritable in the offspring of grafted plants (Cao et al., 2016). The first generation of self-fertilised progenies from tuber mustard (*Brassica juncea*) grafted onto red cabbage (*Brassica oleracea*) displayed a genome-wide change in DNA methylation levels by 5.29% to 6.59% (for hypermethylation and hypomethylation, respectively), compared to the parental lines. By the 5^th^ generation, 31.58% of these changes were still present, while the rest had reverted to the original methylation levels (Cao et al., 2016). Similarly, in tomato and *Arabidopsis*, the vigorous phenotype induced by a mutation in *MutS HOMOLOGUE 1 (*MSH1*)* can be passed through grafting *via* siRNAs, and is inherited in the offspring of wild-type scions (Kundariya et al., 2020).

Over the years, studies have proven that epialleles can arise naturally and can influence agronomically important traits, such as fruit ripening in tomato (Manning et al., 2006) or the seed size and leaf angle of rice (Zhang et al., 2015). Genome-wide and epigenome-wide association studies performed in maize and wheat have highlighted strong correlations between differentially methylated regions (DMRs) and the expression of many genes responsible for crop traits (Gardiner et al., 2018; Xu et al., 2019). These and other studies indicate the great potential of using epialleles in crop breeding for trait improvement. The identification of epialleles linked to agronomically important traits in the progeny of crop grafts could give rise to novel epi-molecular markers (Noshay and Springer, 2021). The prospects of epigenetic breeding have been illustrated in soybean, where improved yields have been observed in the third generation by suppressing MSH1 (Raju et al., 2018). Similar results were observed when replicating MSH1 suppression in tomatoes and *Arabidopsis* (Kundariya et al., 2020). A clear benefit to producing heritable traits through grafting is the possibility of efficiently introducing new genetic and epigenetic diversity from wild species into crops, without the necessity to introgress the trait with breeding or by genetic transformation. Introducing stably transmissible epigenetic traits could ultimately represent a viable and unique technique for developing new crop varieties.

For crop species that are popularly grafted commercially, such as cucumber, tomato, and pepper, future endeavours should identify and preserve the biodiversity of natural varieties and species in light of their potential use as rootstock or scions in grafting combinations. As techniques continue to advance (e.g. micrografting), they will allow us to investigate the genetic and epigenetic variations in the genomes of plants improved by grafting. This will enable the development of new resources and drive comparative studies to fully connect the many facets of epigenetic regulation with the final goal of improving modern crop breeding.

## Author contributions

KJ-C undertook the initial research and wrote the draft of the manuscript, as well as preparing **Figures 1** and **2**. MC provided constructive feedback and novel perspectives, edited the manuscript, introduced new written elements, and finalised the manuscript. All authors contributed to the article and approved the submitted version.
